# In vitro antioxidant effects of barberry fruit extracts

**Published:** 2012

**Authors:** Durdi Qujeq, Solmaz Kamei

**Affiliations:** 1 *Cellular and Molecular Biology Research Center (CMBRC), Babol University of Medical Sciences, Babol, Iran.*; 2 *Department of Biochemistry and Biophysics, Faculty of Medical Sciences, Babol University of Medical Sciences, Babol, Iran.*

**Keywords:** Antioxidant, barberry fruit, aqueous and ethanol extracts, hydroxyl radical

## Abstract

A vast majority of the studies addressing the free radicals including hydroxyl radical is a damage compound of biochemical molecules such as DNA, proteins and lipids. When free radicals specially hydroxyl radical are not adequately removed from the body, it may damage biological macromolecules, leading to a variety of disease occurs. Therefore, the body should be protected by an enzymatic or non-enzymatic antioxidant defense system against free radicals. In order to explore the hypothesis that antioxidant plants can serve as therapeutic agents for diseases, the effect of Barberry fruit extracts was studied in an in vitro model. By evaluating their scavenging potential. Barberry fruits were collected from Babol, Iran and certified by the local scientist Mazandaran Province, Iran. The Barberry fruits were cleaned and dried at room temperature while keeping away from direct sunlight and then powdered. Suitable amounts of dried plant were coarsely grounded and used for extraction. The dry plant samples were extracted with water and/or ethanol. 10 g of Barberry fruits extracts powder was percolated by water for 24 hours. The extract was filtered and concentrated. Hydroxyl radical was produced as described previously. Then, Barberry fruits hydroxyl radical scavenging capacity was determined using deoxyribose degradation system, followed spectrophotometrically at 532 nm. As expected ,our data indicate that the level of hydroxyl radical generation in with aqueous and /or ethanol extracts of barberry fruit was decreased in comparison without barberry fruit extract in vitro system [(6.11±0.83, 5.28 ±1.44, mmol/ml) vs. (9.32±0.38, mmol/ml)], p<0.05, respectively. Indeed, our results revealed that the extracts of the Barberry fruit scavenge hydroxyl radical in vitro sample as compared to the controls. The barberry fruit extracts proved to be an effective for hydroxyl radical scavenging. The present data revealed that beneficial effect of Barberry fruit aqueous and ethanol extracts may be due to its free radical scavenging potential. It may therefore be interesting that he barberry fruit extracts has the unique capacity to quench free radicals.

There is much evidence that over-production of free radicals contribute to biological macromolecules and tissue injury in *in vivo* systems (1-3). According to several studies it is therefore possible that overproduction of free radicals have also been implicated in many diseases (4-6). Earlier studies have suggested that free radicals are constantly formed in the human body and removed by an antioxidant enzymatic and non enzymatic defense system (6). Ther fore an imbalance between reactive oxygen species level and the capacity of antioxidant defenses may cause diseases. It is also well known that reactive oxygen species are generally cytotoxic, because of the oxidative damage they can cause to biological molecules (7-8). At low concentrations, reactive oxygen species may function as physiological mediators of cellular response (9). Oxidative stress, which is associated with the formation of lipid peroxides, contribute to pathological processes in diseases (10). It has also been demonstrated that small amounts of free radicals are constantly generated in aerobic organisms (11-13). The importance of oxidative damage generated under high level of un-scavenged or un-controlled reactive oxygen species was extensively investigated (14-16). Several experimental studies have suggested that an imbalance in antioxidant enzymes was related to many diseases (17-19). 

Few reports in the literature show that scientists are interested in antioxidants and natural products because they could retard the oxidative damage of a tissue by increasing natural defenses (18-19). Up to now, a wide variety of plant has been tested, with varying degree of success. In this regard, Barberry (Berberis vulgaris) is a traditional plant used as an herbal remedy. It does not have any known toxic side effects at reasonable dosages (20). Extracts obtained from the roots of Berberidaceae species have been used as folk medicine in disease and it plays important role in defense system (21). 

Besides this knowledge, it is now clear, that the berberine is isolated as a main alkaloid from the roots and bark of Berberis vulgaris (21). In an attempt to better understand, Its mechanism of aetion the objective of this study was to investigate the barberry fruit extract for its activity as scavenger of hydroxyl radical *in vitro*.

## Materials and Methods

Samples of Barberry fruits were collected from Mazandaran province, Iran.

The Barberry fruits were separated and cleaned, Then dried at room temperature while keeping away from direct sunlight and then powdered. Suitable amounts of dried fruit were coarsely grounded and used for further extraction.

**Preparation of the aqueous extract of Barberry fruits**

In order to prepare the aqueous concentrate, 10 g of the Barberry fruits powder was mixed with 10 ml of distilled water or 10 ml of ethanol for 24-h with continuous stirring . The non soluble part was then separated using mesh. The solution was cooled and filtered. The macerate was filtered and its volume was reduced to 2 ml and consequently kept at -20 ◦C.

**Hydroxyl radical production and scavenging potency**

Hydroxyl radical was produced according to the method of Schinella (22). 0.5 ml extract was added to 0.5 ml of a reaction mixture containing hydroxyl radical and the mixture was shacked. After addition of 0.75 ml of thiobarbituric acid (67%) absorption of samples were detected spectrophotometrically at 532 nm. 

**Statistical analysis**

Statistical data analyses were assessed using Impendent t-test with P<0.05 as the minimal level of significance. Statistical analysis was performed by running the SPSS version 16.0 for Windows package.

## Results

**Fig 1:**
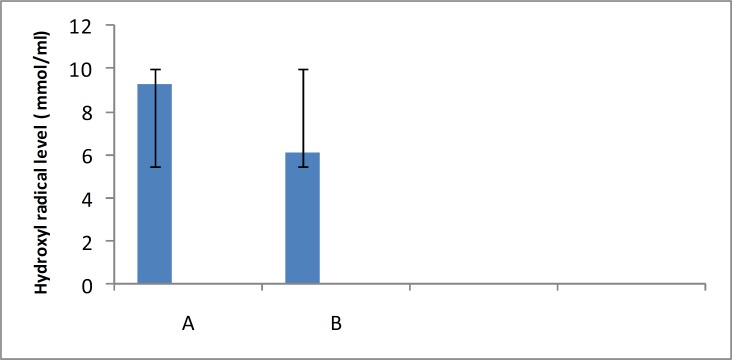
Effects of the aqueous extract of Barberry fruit on hydroxyl radical level in vitro . (A) Without Barberry fruit extract (B) with extract. Each column represents the Mean   SD of 3 separate experiments (P<0.05).

**Fig 2:**
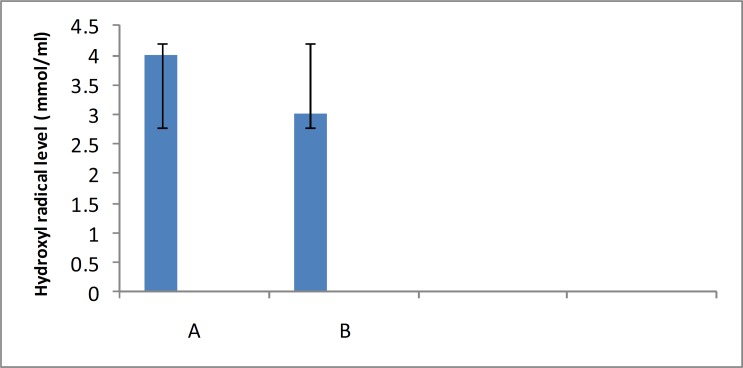
Effects of the ethanol extract of Barberry fruit on hydroxyl radical level in vitro. (A) Without Barberry fruit extract (B) with extract. Each column represents the Mean   SD of 3 separate experiments ( P<0.05).

This study was carried out in order to examine the effect of Barberry fruit extracts for their scavenging capacity of hydroxyl radical. Also, the potential of Barberry fruits extracts to trapping hydroxyl radical was determined in the thiobarbituric acid system and monitored spectrophotometrically. According to this study, aqueous extract of the Barberry fruit protected against hydroxyl radical generation *in vitro*. Scavenging of hydroxyl radical by aqueous extract of barberry fruit was shown in Figure 1. Scavenging of hydroxyl radical by ethanolextract of barberry fruit, was shown in Figure 2.our results clearly demonstrated the capacity of barberry fruit extracts to quench hydroxyl radical.

## Discussion

Antioxidant plant are known to play a major role in defense system. Although the nutritional importance barberry fruit is well known, but the definition of specific effects for barberry fruit has so far proved elusive. The mechanism by which barberry fruit role in health and disease is not well known. Several potential cellular and molecular mechanisms to explain the role of it. Therefore, the resolution of this role needs further basic and clinical experimental investigation.

In this study, aqueous and ethanol extract of Barberry fruit were investigated for their activity as inhibitor and potential trapping of hydroxyl radical *in vitro*. Our results clearly demonstrated that the extracts trapped the hydroxyl radical generation. Our results were in good agreement with those reported previously and confirm earlier data reporting antioxidant effect of barberry fruit (20-21). 

As there was difference between the extracts by using aqueous and ethanol. We found that trapping capacity of methanol extract of Barberry fruit (5.28 ± 1.44, mmol/ml) was higher than aqueous extract (6.11 ± 0.83, mmol/ml). Our results indicate that ethanol extract might be more effective in antioxidant defense system. Our results allow us to suggest that ingestion of Barberry fruit extract might be a very effective and economic way to provide an important amount of compounds that increase the antioxidant defense system. 

Theoretically, antioxidant plant act through a variety of mechanisms such as scavenging free radicals. We hypothesized that the hydroxyl radical trapping property of Barberry fruit aqueous and ethanol extracts might be due to its chemical constitutes. 

The ethanol extract of barberry fruit more protected against hydroxyl radical when compared with water extract. Barberry fruit may be one of the nutritional stimulating factors available for future antioxidant use. But the real role of Barberry fruit extracts upon the defense system merits further experimental and clinical investigations. 

Further experimental investigations also, are needed to delimit the role of Barberry fruit in variety diseases and its therapeutic potential. 

## Notes

**Conflict of intrest:** none dcclared.
